# Lifeact-mEGFP Reveals a Dynamic Apical F-Actin Network in Tip Growing Plant Cells

**DOI:** 10.1371/journal.pone.0005744

**Published:** 2009-05-29

**Authors:** Luis Vidali, Caleb M. Rounds, Peter K. Hepler, Magdalena Bezanilla

**Affiliations:** Department of Biology, University of Massachusetts, Amherst, Massachusetts, United States of America; Purdue University, United States of America

## Abstract

**Background:**

Actin is essential for tip growth in plants. However, imaging actin in live plant cells has heretofore presented challenges. In previous studies, fluorescent probes derived from actin-binding proteins often alter growth, cause actin bundling and fail to resolve actin microfilaments.

**Methodology/Principal Findings:**

In this report we use Lifeact-mEGFP, an actin probe that does not affect the dynamics of actin, to visualize actin in the moss *Physcomitrella patens* and pollen tubes from *Lilium formosanum* and *Nicotiana tobaccum*. Lifeact-mEGFP robustly labels actin microfilaments, particularly in the apex, in both moss protonemata and pollen tubes. Lifeact-mEGFP also labels filamentous actin structures in other moss cell types, including cells of the gametophore.

**Conclusions/Significance:**

Lifeact-mEGFP, when expressed at optimal levels does not alter moss protonemal or pollen tube growth. We suggest that Lifeact-mEGFP represents an exciting new versatile probe for further studies of actin's role in tip growing plant cells.

## Introduction

Plants use tip growth to achieve many essential objectives. For instance, in the moss *Physcomitrella patens*, plant expansion is initially carried out by tip growing protonemata and is thus essential for establishment of the plant [1]. In angiosperms, the pollen grain germinates on the stigma, and then extends a long tip growing tube to deliver the sperm to the ovule. Though the relative importance of various physiological parameters and molecular regulatory pathways involved in tip growth remains controversial, the critical role of actin dynamics in promoting this growth is not.

Studies of actin's role in polarized growth of plant cells have revealed that the dynamic pool of filamentous actin is tightly regulated. Indeed, nanomolar concentrations of the actin depolymerizing drug Latrunculin B disrupt growth without altering cytoplasmic streaming in pollen [2]. In moss as well, Latrunculin B inhibits tip growth [3], [4]. Investigations of the role played by different actin binding proteins in moss, root hairs and pollen tubes have shown that alterations in expression level and regulation dramatically alter tip growth [2]–[13]. These data suggest that the plant cell must maintain a delicate balance between G- and F-actin to promote tip growth. While the actin cytoskeleton's role in polarized growth has been amply demonstrated, it still remains uncertain at a mechanistic level how actin works to promote tip growth. A robust tool for live cell imaging would reveal the structure of the actin network during tip growth and enable detailed studies of the role of actin in tip growth.

Despite many attempts at characterizing actin in live and fixed tip growing plant cells, a consensus has not emerged concerning many features, particularly in the apex. In moss, fixed protonemata show a tip localized collar or aggregation of filaments along with a cortical mesh-work of actin and a network around chloroplasts [11], [13]–[15]. In pollen tubes, the structures yielded by fixation have varied substantially; some studies revealed a dense apical meshwork [16], whereas in others the apical domain was free of actin [2], [17]–[20]. Other work has pointed to a collar around the apical region, but this was not initially seen as a consistent feature [21]. Recently an optimized procedure has revealed the presence of a consistent apical cortical “fringe” in both rapid-freeze and room temperature fixed pollen tubes [22].

Though imaging actin in fixed cells provides a great deal of information, it ultimately yields a static image and is not as useful as live cell imaging. Imaging live-cells allows for the visualization of cytoskeletal structures as they change in response to growth conditions. This, though, has remained difficult. Injecting rhodamine phalloidin into pollen tubes failed to label actin in the extreme apex [23]; subsequent work has shown that the probe is rapidly sequestered into the vacuole. Attempts to express GFP labeled actin have failed largely because pollen tube growth is inhibited [21].

Several different actin binding proteins have been used in an attempt to image actin in growing pollen tubes [24], [25]. Actin depolymerizing factor (ADF) from both lily and tobacco fused to GFP labels actin and does not dramatically alter cell growth in lily or tobacco [5], [24], [25]. However, this probe does not clearly label the apical domain. Mouse Talin (mTalin) [21], [26]–[28], the second actin binding domain of fimbrin [24], and recently a pollen specific LIM protein have also been used to probe actin in live pollen [25]. These probes have shown utility in other cell types, but in pollen the images are inconsistent and do not detect all of the structures shown in the rapidly frozen fixed cells. Specifically, instead of lily pollen's cortical actin fringe, the probes reveal a dense mesh throughout the cell's tip [5], [24], [25].

In moss, attempts to use the actin binding domain of fimbrin fused to GFP have resulted in growth abnormalities and cytoskeletal anomalies (L. Vidali unpublished observations). Although stable expression of mTalin-GFP inhibits cell growth in moss, recent work using a heat inducible promoter upstream of mTalin-GFP did produce labeling of actin. However, the transient nature of the heat-induction only allows for brief imaging of the actin cytoskeleton [4], [29]. In addition, GFP-mTalin has been shown to inhibit tip growth in root hairs [30].

Here we employ Lifeact, a probe first used in animal cells [31], to examine actin in live moss protonemata and pollen tubes. This probe consists of the first 17 amino acids from the budding yeast ABP-140 fused to GFP. In animal cells Lifeact-mEGFP and Lifeact fused to FITC effectively label actin without impairing cell viability [31]. Lifeact peptide fused to FITC has subsequently been used in mouse oocytes to elucidate a novel actin-based mechanism for chromosomal motility [32].

Lifeact-mEGFP allows visualization of actin dynamics in growing moss protonemata and both lily and tobacco pollen tubes. In moss, the probe labels a distinct and consistent apical F-actin network at the growing tip of protonemata with a focal point of F-actin. In lily and tobacco pollen tubes, a highly dynamic apical F-actin network is labeled defining the clear zone. In all three organisms a cortical actin network extends rearward through the cell. In pollen tubes there are also dynamic and distinct interior filaments that appear to be involved in reverse fountain streaming. As a live-cell probe, Lifeact provides a new valuable tool for examining actin organization in tip growing plant cells.

## Results and Discussion

### Lifeact-mEGFP labels a clear three dimensional apical F-actin network in moss and pollen

We analyzed Lifeact-mEGFP labeling of actin in protonemata of the moss *Physcomitrella patens*, a model bryophyte, and pollen tubes from *Lilium formosanum* and *Nicotiana tobaccum*, representing monocots and dicots respectively. These well characterized cells are notable because of their actin dependent, highly polarized tip growth and the ease with which they are transformed.

We constructed a fusion protein consisting of Lifeact [31] fused to mEGFP with a seven amino acid linker (Lifeact-mEGFP). We stably transformed moss with Lifeact-mEGFP under the control of the maize ubiquitin promoter [33]. Several individually transformed lines of moss were isolated and characterized. For lily and tobacco pollen, we performed transient transformations with the same fusion construct under the control of the zmC13 and Lat52 promoters, respectively [34], [35]. To visualize the actin cytoskeleton throughout the cell volume, we used Laser Scanning Confocal Microscopy (LSCM) and recorded z-stacks of live cells expressing Lifeact-mEGFP.

**Figure 1:**
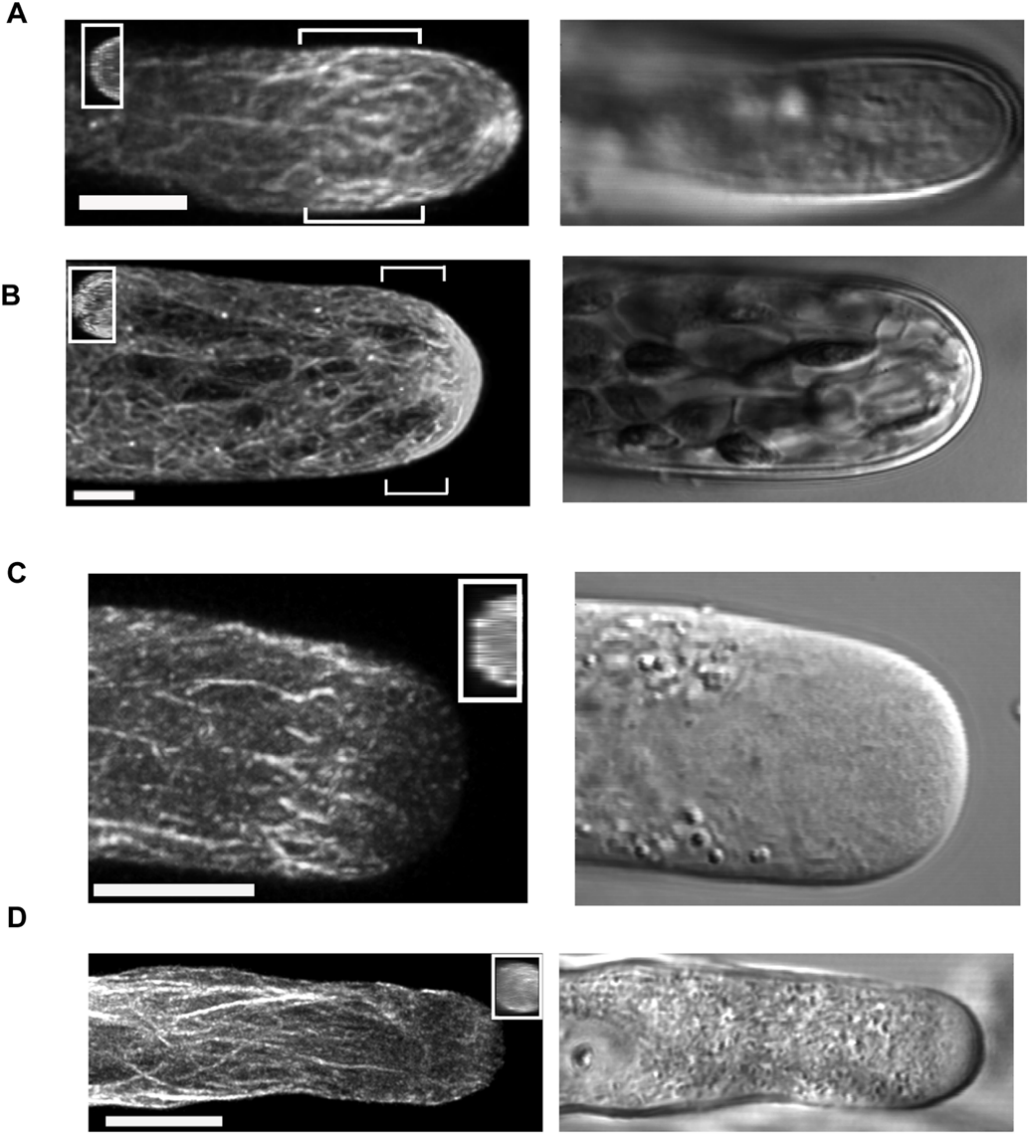
Maximal projections of confocal sections in moss and lily. (A) Maximal projection of a *Physcomitrella* caulonemal cell expressing Lifeact-mEGFP shows a prominent focal point at the apex along with cortical filaments in the subapical region. The insets show the bracketed area rotated 90°. (B) Maximal projection of a *Physcomitrella* chloronemal cell highlights the intense apical signal, which forms a patch with filaments emanating towards the rear of the cell. The insets show the bracketed area rotated 90°. For moss, bars represent 5 µm. (C) A maximal projection of Lifeact-mEGFP signal in lily highlights the cortical actin fringe. Inset shows the same cell rotated to exhibit cortical localization of actin signal at the tip. (D) A maximal projection of Lifeact-mEGFP signal in tobacco shows the longitudinal actin filaments in the shank. Inset shows the same cell rotated 90° highlighting the cortical bias to the apical F-actin network. In both pollen species, the bar represents 10 µm.

Moss protonemata are composed of two cell types: chloronemal and caulonemal cells. Chloronemata contain many chloroplasts, have perpendicular cell plates, and have a poorly defined clear zone. In contrast, caulonemata have fewer chloroplasts, oblique cell plates, a defined clear zone, and grow about three times faster than chloronemata. In previous studies, fixed caulonemal cells have shown an apical actin fringe, but previous live cell imaging, performed with a heat-shock inducible mTalin-GFP construct, did not corroborate these findings [4], [29]. Here, *Physcomitrella* protonemata expressing Lifeact-mEGFP were imaged by collecting serial optical slices in the z axis. In caulonemal cells expressing Lifeact-mEGFP, an apical F-actin network can consistently be visualized near the tip of the growing cell and an actin focal point is also observed (Figure 1A). The brackets define the area rotated and shown in the inset. This highlights the cortical F-actin network consistently seen in caulonemal cells. In chloronemal cells a large amount of F-actin accumulates at the apex, but instead of a small focal point, it appears as a patch (Figure 1B). Filamentous structures are visible within the patch and seem to extend out from it. Towards the back of the cell, the actin microfilaments extend along the cortex. The inset shows the bracketed region of the cell rotated 90°. It demonstrates that although longitudinal fibers exist along the cortex, they are mostly absent from the center of the cell.

Collecting z-stacks of live lily pollen proved challenging as the cells grow rapidly enough that the stack blurs unless collected faster than the cell can grow. To partially alleviate this problem, we took rapid, small images of half the cell's diameter. In lily pollen tubes, a clear fringe, consisting of a palisade of short longitudinally oriented fibers, encircles the cell's apex (Figure 1C). Forward of this fringe, few filaments are evident. The inset shows the same image rotated 90° along the y axis, clearly demonstrating that the Lifeact-mEGFP signal is largely cortical, with reduced signal in the middle of the tube. Significantly, Lifeact-mEGFP labels both G- and F-actin, so some of the signal in the center of the tube may be G-actin [31]. However, we routinely observed some microfilaments (F-actin) in the center of the tube. These images are consistent with the data from rapidly frozen fixed cells [22].

In tobacco pollen, Lifeact-mEGFP labels longitudinal fibers along the shank of the tube (Figure 1D). An apical F-actin network is also present though it is closer to the tip and more dense in the medial planes than the fringe seen in the lily pollen tube. The inset shows the same image rotated 90°. It highlights the apical F-actin network. Although some F-actin is located in the center of the tube, a great deal is positioned in the cortex. Lifeact-mEGFP confirms the existence of a fringe in lily as shown in the rapidly frozen cells [22], and shows that a similar structure exists in tobacco. Taken together, these data demonstrate the existence of an apical F-actin network in all three cell types.

### Lifeact-mEGFP and phalloidin label the same structures in MBS-EGS fixed moss cells

**Figure 2:**
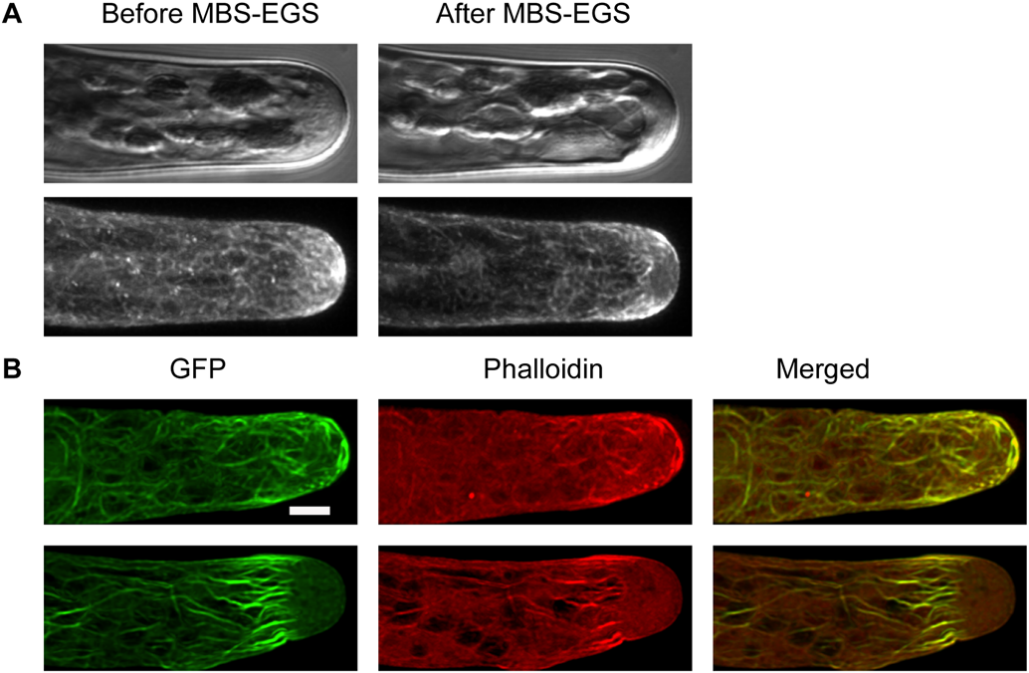
Lifeact-mEGFP and phalloidin label the same structures in MBS-EGS fixed moss. (A) shows a cell before and after fixation. Top is DIC, bottom is GFP signal. An apparent change in actin localization has occurred. (B) Two different cells expressing Lifeact-mEGFP (left) stained with rhodamine phalloidin (middle). Far right panels show the merged signal. Bar is 5 µm.

Our results with Lifeact-mEGFP were not entirely consistent with recent work using fluorescently conjugated phalloidin to label actin microfilaments in moss [11], [13]. Though the network labeled by Lifeact-mEGFP is similar in some respects to that seen in the fixed-cell images, there are some differences. We wished to investigate whether the difference between these two F-actin patterns is due to fixation, or whether Lifeact-mEGFP and fluorescently conjugated phalloidin are labeling distinct structures. We began by monitoring Lifeact-mEGFP before and after cross-linking with MBS-EGS (Figure 2A). Clearly some rearrangements in the Lifeact-mEGFP pattern occur; most importantly, the tip localized focal point dissipates and in general the filaments seem thicker as if bundling has occurred.

To verify that Lifeact-mEGFP and phalloidin label the same structures, we used MBS-EGS to cross-link cells expressing Lifeact-mEGFP. After cross-linking, we fixed the cells and processed them for labeling with rhodamine-phallodin. We then examined the localization of the two probes (Figure 2B). Significantly, the probes co-localize throughout the cell, both in cells that have an apical F-actin network and those that lack apical labeling (compare top and bottom frames in Figure 2B). These data demonstrate that Lifeact-mEGFP is labeling the same F-actin as labeled by rhodamine phalloidin in fixed cells. They also suggest that MBS-EGS fixation alters the localization of the apical F-actin network at the extreme apex, causing a loss of signal at the tip and bundling of filaments towards the rear of the cell. In particular, fixation enhances labeling of the fringe-like structure in the apex of some caulonemal cells. Lifeact-mEGFP circumvents these artifacts, allowing labeling of actin in living cells.

### Lifeact-mEGFP reveals actin dynamics during growth

**Figure 3:**
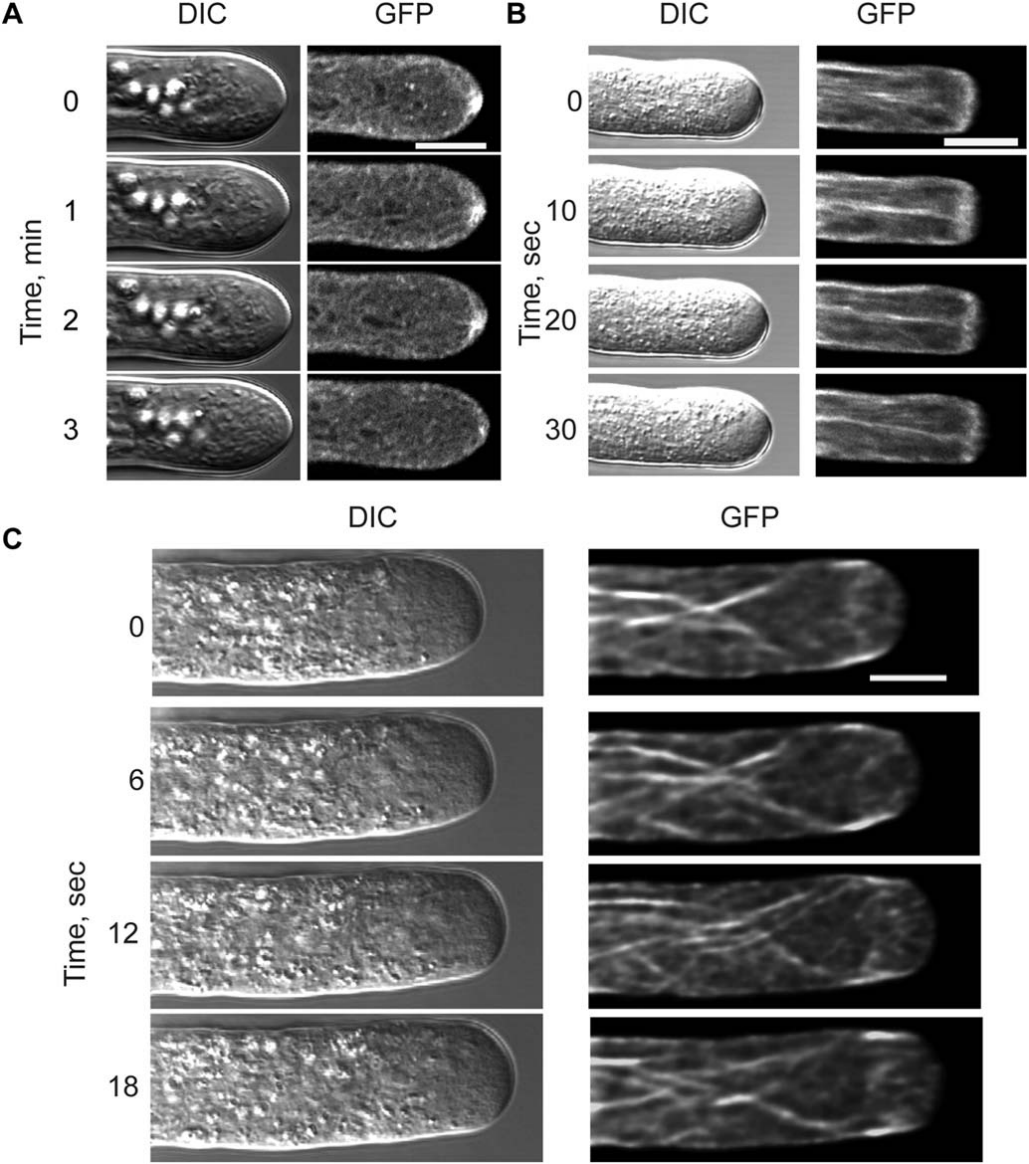
Lifeact-mEGFP labels dynamic actin in moss and pollen. (A) Confocal micrographs of *Physcomitrella patens* showing the actin focal point at the apex. (B) The presence of the apical F-actin network as seen in *Nicotiana tobaccum*. (C) The cortical fringe in the apex of a *Lilium formosanum* pollen tube expressing Lifeact-mEGFP. Bar is 10 µm. See Movies S1, S2 and S3 for complete series.

To image the remodeling of actin filaments and the apical F-actin network in growing cells, we collected time lapse, medial plane images with LSCM. Lifeact-mEGFP labels dynamic filamentous structures throughout moss caulonemal cells. Significantly, in the medial plane a distinct focal point of actin is seen at the cell apex (Figure 3A). In the full movie from which these stills are taken (Movie S1), one can see actin filaments radiating out from this focal point. The still images, shown at 1 minute intervals, highlight the highly dynamic actin filaments at the cell apex (Figure 3A). Interestingly, although an apical focal point of actin is visible in all growing tip cells, its localization varies. Sometimes it is at the extreme tip, while at other times it is to the side. Images using mTalin-GFP have also noted apical accumulations of actin, although with less distinct filamentous structure [4], [29].

In *Nicotiana tobaccum* pollen tubes, Lifeact-mEGFP labels longitudinal filaments as well as a dense apical F-actin network in the medial plane (Figure 3B). Images are shown at 10 second intervals. Notably, the apical F-actin network is not stationary; it varies in its exact distance from the tip. However, it does maintain its position relative to the clear zone of the pollen tube. In the complete movie it is apparent that short filaments are constantly moving in and out of the center of the apex (see Movie S2).

In culture, the larger lily pollen grows much more rapidly than either tobacco or moss. Figure 3C shows medial plane images taken at 6 second intervals from a growing lily pollen tube. The cortical actin fringe is observed along the sides of the clear zone, as seen in fixed cells [22]. In addition, there are some microfilaments in the apical core that are constantly being remodeled and occasionally swept rearward (see Movie S3). Furthermore a funnel-like structure appears to taper backwards from the fringe, also consistent with previous studies [2].

These data demonstrate that Lifeact-mEGFP labels F-actin structures and enables imaging of the rapid remodeling of the actin cytoskeleton in a growing tip cell. Lifeact-mEGFP confirms the presence of a cortical actin fringe in lily in keeping with what has been shown in rapid-freeze fixed pollen tube cells probed with anti-actin antibodies [22]. It also shows a dynamic apical F-actin network and many longitudinal fibers. These observations differ from those generated through the use of GFP-ntADF1 and ntLIM2b-GFP [25], which do not clearly resolve apical filamentous structures, in particular the cortical actin fringe in the apex of lily pollen tubes.

### Rapid actin remodeling occurs at the tip region in moss

**Figure 4:**
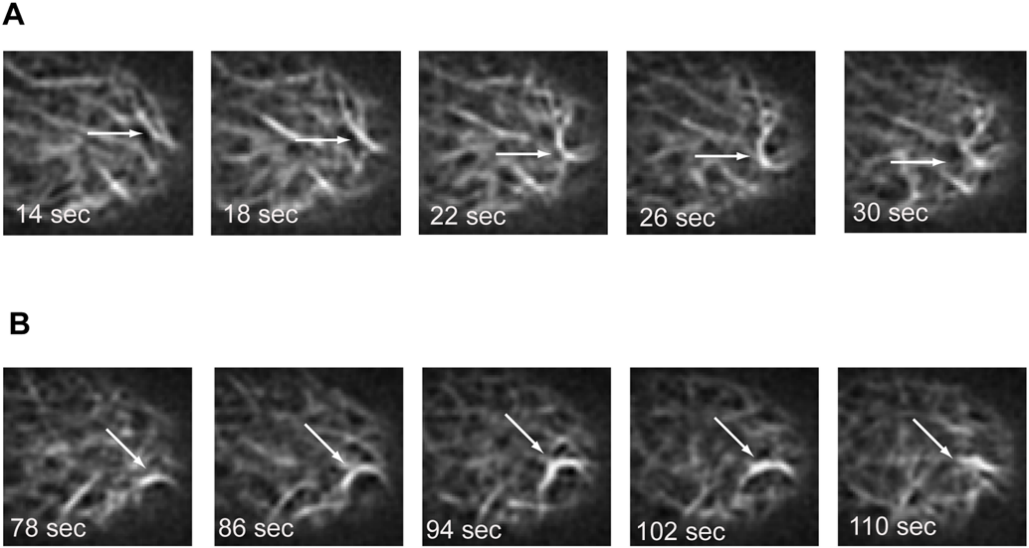
Spinning disc confocal images show possible actin buckling at the tip region in moss. (A) and (B) show a moss cell expressing Lifeact-mEGFP imaged at times indicated. Arrows point to potential buckling event as seen in successive planes. See Movie S4 for complete series.

Our time lapse imaging of Lifeact-mEGFP showed changes in the actin network's structure that were surprisingly fast. To capture these changes, we imaged Lifeact-mEGFP in moss with a spinning disc confocal instrument. This instrument scans the frame 360 times a second, instead of scanning through the frame once or twice a second. It thus eliminates some of the blurring caused by the scan of the conventional confocal instrument. This allows for dramatic, rapid alterations in actin to be more accurately imaged. We acquired a time lapse series of cortical slices in a growing cell (Figure 4). The images show the rapid remodeling of microfilaments occurring in the cortex at the cell tip (Movie S4). Arrows point to possible buckling events seen very close to the apex. These results are very similar to recent studies from imaging of actin in Arabidopsis epidermal cells using variable angle epifluorescence microscopy [36]. Buckling events have been described before *in vitro*. Actin immobilized by myosin at one end then elongated by formin at the other is forced to buckle, generating significant force [37]. From the Lifeact-mEGFP images it is of course impossible to infer the proteins involved in the event, nor can we conclude that these structures are producing the same forces as seen in the *in vitro* experiment. However, these images present compelling evidence that buckling events might be playing a role in polarized tip growth in moss.

### High expression of Lifeact-mEGFP affects moss protonemal growth

As many live cell actin probes significantly inhibit growth, we investigated whether Lifeact-mEGFP affects growth in moss protonemata or pollen tubes [5], [24]. In moss we characterized Lifeact-mEGFP in two distinct genetic backgrounds: WT and NLS-4. NLS-4, a line important for RNAi based loss-of-function studies, is a stable transgenic line that expresses a GFP-GUS fusion with a nuclear localization signal [11], [13], [38]. We obtained several independent lines and analyzed the expression and growth in both backgrounds.

**Figure 5:**
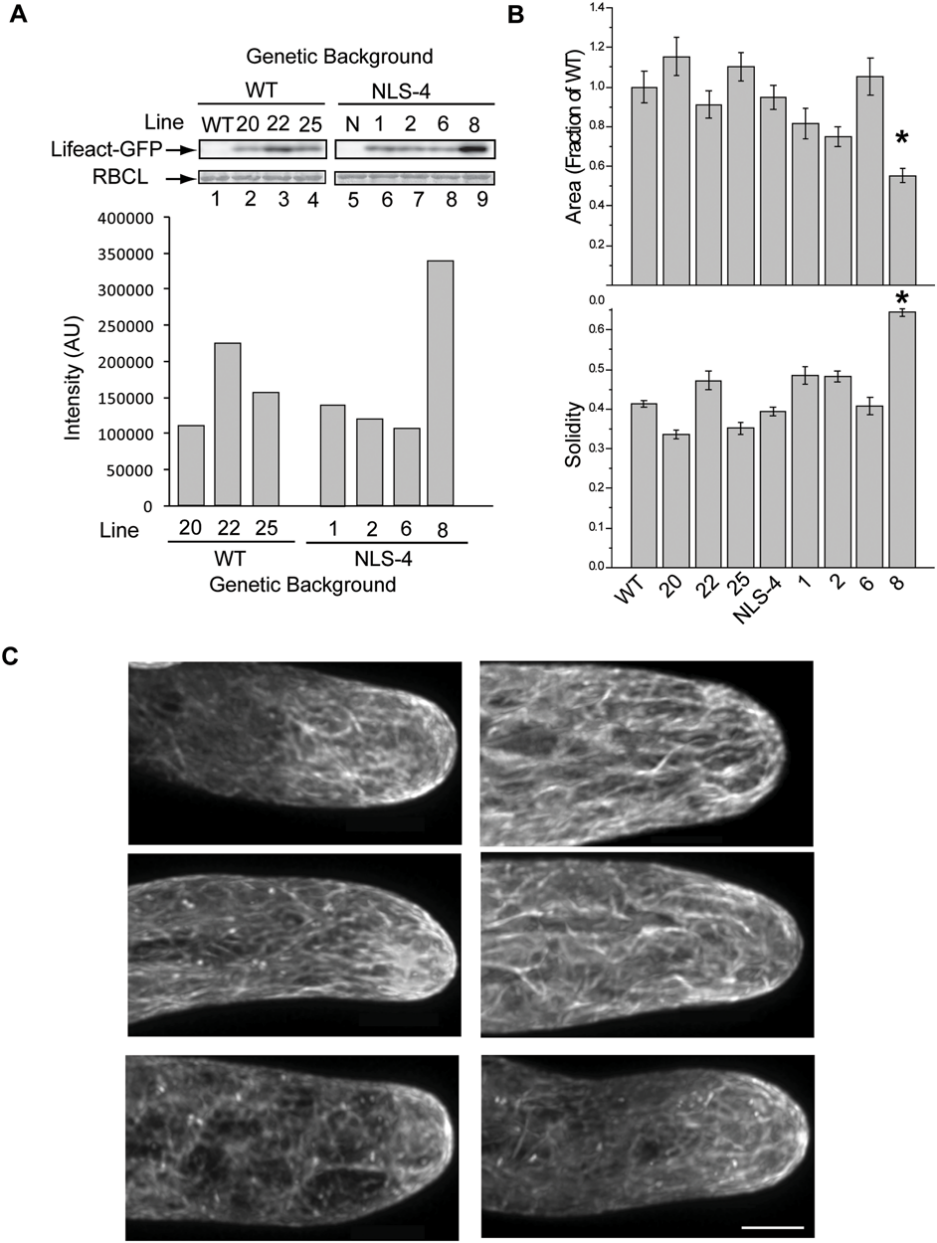
High expression of Lifeact-mEGFP affects growth in moss protonemata. (A) Top panel shows protein extracted from indicated moss lines resolved by SDS-PAGE then immunoblotted with GFP specific antibodies. Coomassie stained RUBISCO large subunit (RBCL) is shown as loading control. The lower panel displays relative quantitation of immunoblot band intensity shown in top panel. (B) shows the results of a growth assay performed upon moss stably transformed with Lifeact-mEGFP lines. Top panel shows plant area as a fraction of the WT. Bottom panel shows solidity. Line 8 indicated by the asterisk is significantly different from WT and NLS4 for both area and solidity (ANOVA p<0.05). Variations in area for all other lines are not significant. (C) Three representative maximal projections of tip cells from line 22 (left) and line 8 (right). Bar is 5 µm.

We isolated protein from seven day old protonemata and used immunoblotting to evaluate the relative amount of Lifeact-mEGFP expressed in each line. In the WT background, line 22 expresses twice as much Lifeact-mEGFP as line 20 and nearly half again as much as line 25 (Figure 5A compare lanes 2, 3 and 4). In the NLS-4 background, line 8 expresses 3 fold more than the lowest expressing line (Figure 5A compare lanes 8 and 9). To determine whether the amount of Lifeact-mEGFP expressed affects plant growth, we examined the growth properties of the seven Lifeact-mEGFP lines. Young moss plants regenerating from protoplasts are composed exclusively of protonemal tissue and one can measure growth by comparing the area of individual plants. We stained plants with the fluorescent dye calcofluor, and used the signal to calculate the area of individual plants. Additionally we used solidity, which is the area divided by the convex hull area as an indication of overall filamentous outgrowth (see Methods). Solidity values approaching one indicate that the plants are solid and lack polarized extensions; lower values indicate the presence of filamentous outgrowths and a higher degree of plant polarization [11]. Line 8 in the NLS-4 background results in smaller plants indicating the slowest growth rate and a concomitant increase in solidity (Figure 5B). This line has the highest level of Lifeact-mEGFP expression. All the other Lifeact-mEGFP lines have similar areas as compared to wildtype or the NLS-4 control. Line 8 appears to produce far fewer caulonemal cells as compared to controls, presumably contributing to the increase in solidity. High levels of GFP do not exhibit these growth defects (data not shown). Interestingly, neither line 8 nor line 22, the two lines expressing the highest levels of Lifeact-mEGFP, exhibited dramatic actin artifacts (Figure 5C for representative images). In contrast, many other actin binding protein probes have been shown to create circles and large bundles when over expressed [24], [25]. The Lifeact-mEGFP labeling looks similar to lines with lower levels of expression, although with a higher diffuse cytoplasmic signal (compare to Figure 1). Our data indicate that Lifeact-mEGFP expression levels that allow effective imaging in live cells allow normal cell growth characteristics.

Lifeact-mEGFP thus presents several advantages for studying the actin cytoskeleton in moss. It avoids potential artifacts arising from fixation, particularly from the cross-linking; it does not inhibit filamentous or bulk growth; and expression is constant, so that no manipulations are necessary to allow for imaging. Significantly, Lifeact-mEGFP is expressed in a stable line presenting the opportunity for studying other structures and organelles concurrently using different probes.

### Lifeact-mEGFP and GFP alone have equivalent affects on pollen tube growth

**Figure 6:**
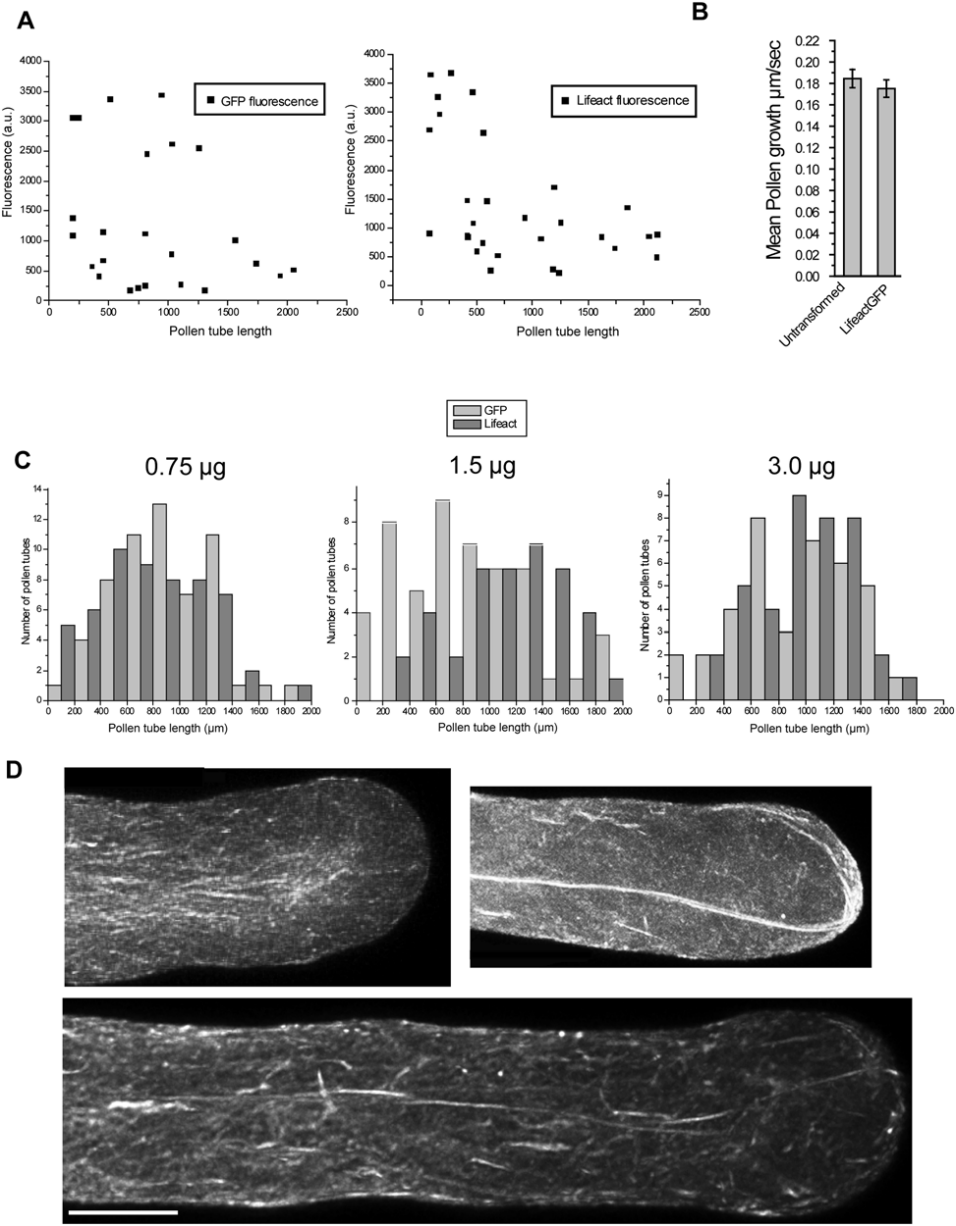
GFP and Lifeact-mEGFP have equivalent effects on lily pollen tube growth. (A) Lily pollen was transformed with 3.0 µg of GFP or Lifeact-mEGFP, imaged, then average fluorescence per pixel and pollen tube length were measured. (B) Lily pollen transformed with Lifeact-mEGFP. DIC image series of at least 4 minutes were collected of transformed and untransformed pollen on the same slide. Error bars indicate standard error (n = 9 for untransformed and n = 12 for transformed, t-test p = 0.4472). (C) Pollen transformed with increasing amounts of DNA of both GFP (light grey) and Lifeact-mEGFP (dark grey) were fixed after three hours, imaged and measured. No difference in length distribution is apparent. (D) Representative images of pollen tubes expressing Lifeact-mEGFP that have stopped growing, but still exhibit cytoplasmic streaming.

Previous studies with actin binding probes have shown that the amount of DNA used to transform pollen affects both expression level and growth rates [5], [24], [25]. To determine if high expression levels of Lifeact-mEGFP correlated with mortality, we compared growth in pollen transformed with either 3 µg of plasmid encoding Lifeact-mEGFP or GFP alone under the control of the zmC13 pollen specific promoter. We imaged transformed pollen tubes after allowing them to grow for three hours. From these data, we measured the length and the average fluorescence per pixel in each pollen tube (Figure 6A). Though the highest expressing tubes tend to be shorter, this is true for both GFP and Lifeact-mEGFP. This shows that both GFP and Lifeact-mEGFP at high expression levels reduce growth, suggesting that high levels of Lifeact-mEGFP are no more toxic than high levels of GFP.

We wished to compare growth of transformed pollen tubes to untransformed pollen tubes. To this end we transformed pollen with Lifeact-mEGFP and collected high-resolution images of transformed and untransformed cells. We then tracked the growth of 10 individual cells of both types for comparison (Figure 6B). Once again, Lifeact-mEGFP transformed cells exhibit a growth rate that is not significantly different from untransformed pollen tubes.

To address whether Lifeact-mEGFP negatively affects growth, we transformed pollen with increasing amounts of Lifeact-mEGFP and GFP plasmid DNA from 0.75 µg to 3.0 µg. To ensure that the length of pollen tubes did not change over the course of the experiment, we fixed the pollen tubes in 3.7% formaldehyde for 30 minutes prior to imaging [2]. The pollen tube lengths were then measured. At all amounts of DNA, GFP and Lifeact-mEGFP showed very similar distributions of pollen tube length (Figure 6C). These findings further validate that Lifeact-mEGFP is no more toxic to the pollen tube than GFP. To investigate whether Lifeact-mEGFP was altering actin dynamics, we collected high resolution z-stack images of pollen that exhibited cytoplasmic streaming but lacked growth. At no time did we observe the dramatic actin artifacts seen with other GFP labeled actin binding probes (Figure 6D) [24], [25]. The major difference between these pollen tubes and the pollen that was growing well, is the lack of a cortical actin fringe. As the length of Lifeact-mEGFP transformed pollen tubes was equivalent to pollen tubes expressing GFP alone, it seems unlikely that alteration in actin dynamics caused these pollen tubes to grow poorly.

These data indicate that Lifeact-mEGFP can be used to reliably label actin in growing cells. Alterations to growth due to of high levels of Lifeact-mEGFP appear to be dependent on the species, with moss more sensitive than lily pollen. Nevertheless, Lifeact-mEGFP's robust labeling suggests that it will provide a tool for visualizing actin while imaging structures, organelles, or ions. Similarly, it will serve as an excellent tool for studying the changes in actin structure as growth oscillates and as manipulations to the cell's environment alter growth.

### Latrunculin B induced actin depolymerization and disrupted filamentous localization of Lifeact-mEGFP signal

The depolymerizing drug Latrunculin B has been widely used to investigate actin's role in various cell processes. For example, nanomolar concentrations abolish growth in pollen tubes but do not alter cytoplasmic streaming [2], [39]. Experiments in moss have also demonstrated sensitivity to Latrunculin B, though at micromolar concentrations in multi-day experiments [3], [4]. As Lifeact-mEGFP appears to be labeling actin faithfully, we sought to investigate whether its localization would be altered by Latrunculin B treatment. Specifically, we predicted that concentrations of Latrunculin B that inhibit growth would dissipate the apical F-actin network.

**Figure 7:**
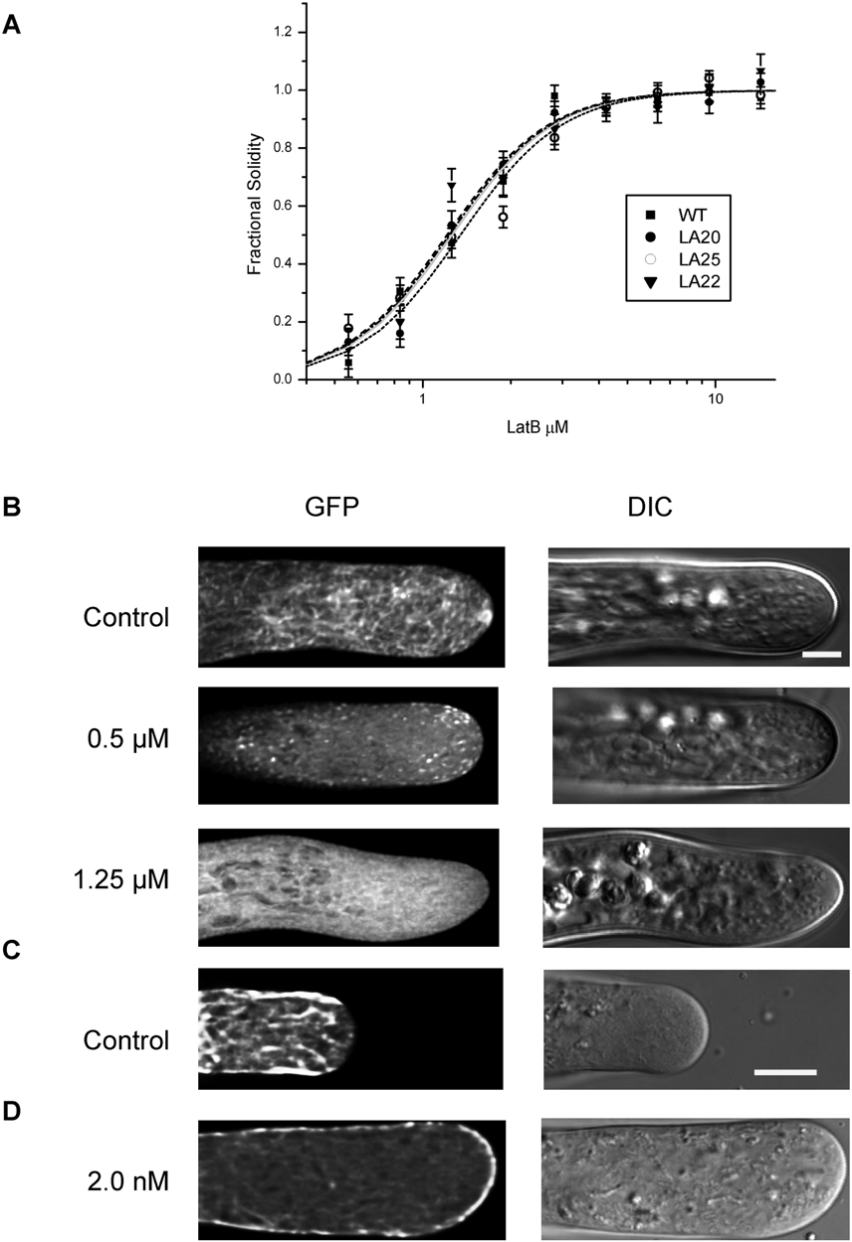
Latrunculin B-induced actin depolymerization alters localization of Lifeact-mEGFP signal. (A) Regenerating moss protoplasts were transferred to media containing increasing concentrations of Latrunculin B. After two days, solidity was measured and plotted versus the log of the concentration. The following are the IC50 values in µM calculated from these data: WT = 1.21±0.07, Line 20 = 1.26±0.03, Line 22 = 1.23±0.12, Line 25 = 1.34±0.06. (A total of 21–115 plants was analyzed per data point. ANOVA statistical analysis shows no significant differences.) (B) Moss cells expressing Lifeact-mEGFP, which have been subjected to increasing concentrations of Latrunculin B no longer show filamentous structures (bottom panel). Bar is 5 µm. (C) Lily pollen growing in control media reveals the cortical actin fringe. (D) After treatment with two 2 nM Latrunculin B, the filamentous signal has been lost and the fluorescence is now mostly cortical. Bar is 10 µm. See Movies S5 and S6 for complete series.

As a first step we examined the effect of Latrunculin B on moss protonemata by comparing the growth of WT plants to the three Lifeact-mEGFP lines in the WT background. Lifeact-mEGFP and WT plants were regenerated from single protoplasts. After four days, the protoplasts were transferred to media containing increasing concentrations of Latrunculin B. Two days after incubation in Latrunculin B, whole plants were imaged to determine plant area and solidity (see Methods). Increasing concentrations of Latrunculin B inhibits tip growth, which is represented by an increase in plant solidity. All assayed lines, including lines 22 and 25, which contain 2- and 1.5-fold more Lifeact-mEGFP respectively (Figure 5A), had similar IC50 for Latrunculin B (Figure 7A). This is particularly significant because it suggests that actin is not stabilized by increasing amounts of the Lifeact-mEGFP probe, consistent with previous *in vitro* results [31].

To examine the effects of Latrunculin B on actin localization as visualized by Lifeact-mEGFP, we transferred Lifeact-mEGFP expressing plants to agar pads containing DMSO, 0.5 µM or 1.25 µM Latrunculin B. Cells were then imaged after 10 minutes. Cells in DMSO showed no alteration in either growth or Lifeact-mEGFP signal (Figure 7B and data not shown). Cells imaged from the 0.5 µM treatment had stopped growing. They also showed fewer actin filaments, and manifested punctate fluorescence suggesting F-actin depolymerization. The cells incubated in 1.25 µM Latrunculin B also stopped growing and lost any clear actin localization; instead the fluorescence was diffuse throughout the cytoplasm.

To analyze Latrunculin B treatment on pollen tubes, we transformed lily pollen with Lifeact-mEGFP and allowed it to grow for two hours in standard media. After imaging selected cells for several minutes, we replaced the growth media with media supplemented with 2 nM Latrunculin B. This concentration has been used in the past to reversibly terminate growth [2], [39]. We then collected a time lapse image series of the growing pollen tube as it reacted to the drug. Before treatment, the clear zone is apparent and the fringe appears as cortical brightness in a medial plane view (Figure 7C, representative image). Figure 7D shows the same tube after growth has stopped. In line with previous results, the clear zone has collapsed and the tip has swollen. Cytoplasmic streaming continues, but it is no longer organized into the typical reverse fountain [2] (Movies S5 and S6). The fringe has dissipated. Some actin filaments remain, but they are disorganized and largely cortical (Movie S6). Imaging multiple planes in the z-axis reveals filamentous staining in the cortex, but the microfilaments appear to be randomly oriented (Figure S1). These data indicate that the apical F-actin network is important for growth.

Our results indicate that Lifeact-mEGFP expressing moss protonemata and pollen tubes both respond to Latrunculin B treatment. In moss, protonemal cells cease growing and filamentous Lifeact-mEGFP fluorescence is lost. In lily pollen tubes, Lifeact-mEGFP fluorescence is reduced, the tubes stop growing and the tip swells. The tremendous difference in sensitivity to the drug likely results from the wide variance in growth rates; pollen tubes endocytose at a fast pace [40] and therefore will take up a great deal of the drug quickly, whereas the slowly growing moss may take it up more slowly.

### Lifeact-mEGFP robustly labels actin in moss subapical protonemal and gametophore cells

**Figure 8:**
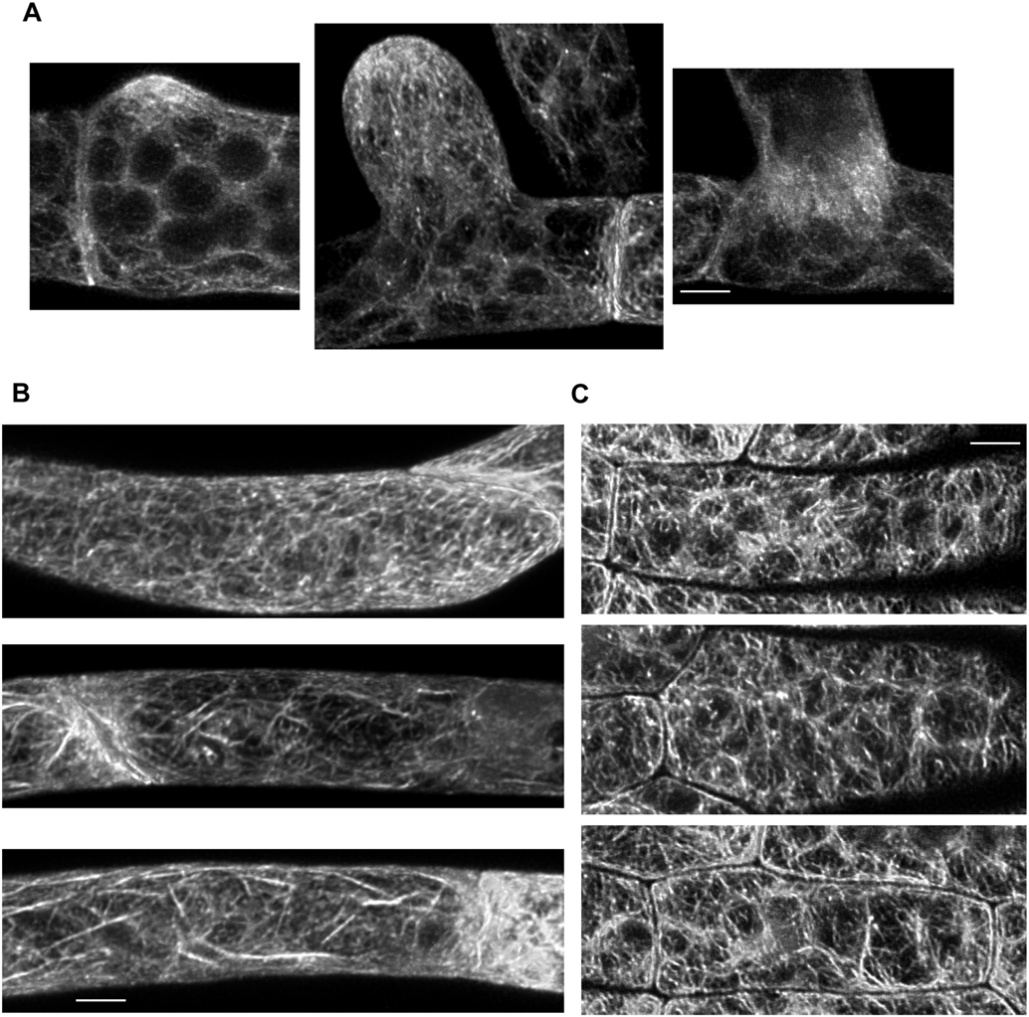
Maximal projections of Lifeact-mEGFP labeling in moss subapical protonemal and gametophore cells. (A) shows a sequence of maximal projections of z-stacks documenting branch emergence from a moss chloronemal cell expressing Lifeact-mEGFP. (B) shows representative subapical caulonemal cells expressing Lifeact-mEGFP. (C) Representative gametophores cells expressing Lifeact-mEGFP. Bars are 5 µm.

As the moss is stably transformed, we were able to monitor actin labeling in a variety of different cell types using line 25. We took advantage of this to examine branch formation in a chloronemal cell (Figure 8A). As the branch begins to emerge, a focal point of actin develops at the tip. Behind this, the actin caging around the chloroplasts is clearly evident. This focal point increases in size as the branch lengthens, resembling the apical actin structure observed in chloronemal cells at the apex of a filament. Finally, a phragmoplast forms at the cell junction (Figure 8A). We collected z-stacks of subapical caulonemal cells. Not surprisingly, the cages around chloroplasts are less prominent as these cells have many fewer chloroplasts. However longitudinal cortical filaments are evident as is a striking accumulation of Lifeact-mEGFP at the cell plate. We also examined cells in gametophore leaflets. Labeling of cortical microfilaments is apparent as is caging around the chloroplasts. Taken together these images demonstrate that Lifeact-mEGFP enables live imaging of actin in a variety of cell types.

### Conclusions

In this report we use Lifeact-mEGFP as a live cell probe for actin in the moss *Physcomitrella patens* as well as in pollen from two species, *Lilium formasanum* and *Nicotiana tobaccum*. Our data indicate that Lifeact-mEGFP possesses significant advantages in tip growing cells over other commonly used live-cell probes. At moderate levels of expression, Lifeact-mEGFP does not inhibit growth in moss and because of this, there is no need to induce expression of the probe; it can be expressed from a constitutive promoter. In moss Lifeact-mEGFP highlights an apical patch of actin filaments in chloronemal cells and a focal point of F-actin in caulonemal cells. These apical networks appear to be areas of intense actin filament production. In pollen tubes, the probe does not retard tip growth. In lily pollen, Lifeact-mEGFP fluorescence compellingly supports the presence of a cortical actin fringe as shown in rapidly frozen and fixed cells [22]. The images are more consistent and the signal to noise ratio is higher than seen with other probes in live pollen tubes [24]–[26]. Tobacco cells also exhibit a variable apical network as they grow. Interestingly, in both pollen species the apical F-actin network is seen to define the edge of the clear zone. The apical F-actin networks in all three species are constantly changing during growth, manifesting the role of dynamic actin in growth. Most significantly, Lifeact-mEGFP will serve as a useful tool for studying the role of actin in living tip growing cells, thus allowing for a much more complete analysis of the factors, both physiological and molecular, involved in tip growth.

## Methods

### Constructs and Stable Line Construction

pTH-Ubi-Lifeact-mEGFP was constructed via multi-site gateway (Invitrogen). Entry clones containing the Lifeact peptide and mEGFP were generated via BP clonase from PCR products. For Lifeact, the first 51 bp of the coding sequence of the ABP140 gene were amplified from yeast genomic DNA, using primers: LifeactB1F-GGGGACAAGTTTGTACAAAAAAGCAGGCTTAATGGGTGTCGCAGATTTG, and LifeactB5rR-GGGGACAACTTTTGTATACAAAGTTGTTTCTTCCTTTGAGATGCTTTC. For mEGFP, we used primers: attB2-mEGFP-STOP-r-GGGGACCACTTTGTACAAGAAAGCTGGGTATTACTTGTACAGCTCGTCCATGCC and attB5-mEGFP-STOP-f-GGGGACAACTTTGTATACAAAAGTTGTGGTGAGCAAGGGCGAGGAG. The entry clones generated by the BP clonase reaction were sequenced and cloned together into pTH-Ubi-Gate [2] via LR clonase. The resulting expression construct was verified by restriction digest. For stable transformation, plasmids were digested with SwaI and transformed using standard procedures [13]. Stable plants were identified by the resistance to hygromycin, after periods of release from selection.

Lifeact-mEGFP was amplified out of pTH-Ubi-Lifeact-mEGFP using the sense primer GGGGGATCCATGGGTGTCGCAGATTTGAT and the anti-sense primer CACGTCGACTTACTTGTACAGCTCGTCC. For tobacco pollen expression, the fragment was then digested with Bam HI and Sal I and inserted into a modified pBS SKII+ that includes the Lat52 promoter [34]. For lily expression, the same construct was subcloned into pBS SKII+ under the control of the zmC13 promoter using the same enzymes [35].

### Bombardment

Plasmid DNA was prepared using alkaline lysis followed by precipitation with PEG and extraction with phenol-chloroform. DNA was coated onto 1 to 3 mg of 1.1 µm diameter tungsten particles (Bio-Rad Laboratories) according to the manufacturer's instructions. The coated microprojectiles were aliquoted onto two macrocarriers (Bio-Rad Laboratories). Pollen was allowed to hydrate in 1 mL of the appropriate growth media (see below) before being placed on a 25 mm MF-Millipore membrane (Millipore), which in turn was set on Whatmann paper moistened with pollen growth media. The macrocarrier assembly was positioned in the top slot of the PDS-1000/He biolistic system and the sample assembly in the slot below (Bio-Rad). Pollen grains were bombarded twice (once with each aliquot) using an 1100-psi rupture disc (Bio-Rad). After bombardment, pollen was transferred to a microcentrifuge tube and incubated for 2 hours at room temperature with constant rotation. Cells were then immobilized on a microscope slide in growth media supplemented with 1.4% low-melting point agarose and imaged 1–4 hours later.

### Pollen/Moss Culture Conditions

For high-resolution imaging, protonemata were subcultured on moss NO_3_ medium, PpNO_3_ (1.03 mM MgSO_4_, 1.86 mM KH_2_PO_4_, 3.3 mM Ca(NO_3_)_2_, 45 µM FeSO_4_, 9.93 µM H_3_BO_3_, 220 nM CuSO_4_, 1.966 µM MnCl_2_, 231 nM CoCl_2_, 191 nM ZnSO_4_, 169 nM KI, 103 nM Na_2_MoO_4_) for at least three days before transfer to an imaging chamber. Protonemata were placed on a 1% agar pad in Hoagland's medium (4 mM KNO_3_, 2 mM KH_2_PO_4_, 1 mM Ca(NO_3_)_2_, 89 µM Fe citrate, 300 µM MgSO_4_, 9.93 µM H_3_BO_3_, 220 nM CuSO_4_, 1.966 µM MnCl_2_, 231 nM CoCl_2_, 191 nM ZnSO_4_, 169 nM KI, 103 nM Na_2_MoO_4_, 1% sucrose), covered with a glass coverslip, sealed with VALAP (1∶1∶1 parts of vaseline, lanoline and paraffin) and immediately observed. Bleaching and cell damage were minimized by using low laser levels (1–2%).

All pollen was grown from frozen stocks (−80°C) collected from plants grown under standard greenhouse conditions. *Lilium formosanum* pollen was germinated and cultured in growth medium consisting of 7% (w/v) Sucrose, 1 mM KCl, 1.6 mM H_3_BO_3_, and 15 mM MES buffer adjusted to pH 5.5 with KOH (LPGM). *Nicotiana tabacum* (cv Petit Havana SR1) pollen was germinated and cultured in medium consisting of 20 mM MES, 0.07% Ca(NO_3_)_2_ tetrahydrate, 0.02% MgSO_4_, 0.01% H_3_BO_3_, 0.01% KNO_3_ and 7% sucrose adjusted to pH 6. Pollen was germinated and grown on a rotor at room temperature. For microscopic observations, a pollen suspension was plated and immobilized with a growth medium solution containing a final concentration of 0.7% (w/v) low-melting agarose. The immobilized pollen was then covered with growth media for imaging.

### Microscopy

Images were collected using the 488 nm argon laser of a Nikon confocal microscope (Nikon D-Eclipse-C1) on an inverted stand (Nikon Eclipse-TE2000-S) using a 60× oil immersion 1.4-numerical aperture objective, a 40× oil immersion 1.3-numerical aperture objective, or a 60× water immersion 1.2-numerical aperture objective. Spinning disc confocal images were acquired with a Perkin Elmer confocal box and an OrcaER CCD camera on a Nikon inverted stand with a 100× oil immersion 1.4-numerical aperture objective.

### Protein extraction and immunoblotting

Moss protein was extracted from previously frozen tissue that was immersed in liquid nitrogen prior to extraction. The tissue was first homogenized in liquid nitrogen, then grinding buffer (100 mM Na_2_PO_4_ pH 7.0, 10 mM DTT, 20% glycerol and 0.1% protease inhibitor cocktail (P9599 Sigma))was added and the resulting slurry was further homogenized. The slurry was then subjected to centrifugation for 10 minutes in a benchtop microfuge. The resulting extract was separated by SDS-PAGE, transferred to a nitrocellulose membrane and immunoblotted with anti-sera to GFP (Invitrogen).

### Image Processing

Image processing was performed with AutoDeblurGold Cf (MediaCybernetics) using 5–30 three-dimensional deconvolution iterations and displayed as a maximal z-projection for z-sections. For image sequences, two dimensional blind deconvolution was performed using 5–30 iterations. Subsequent deblurring was performed with the same software.

### Moss Fixation

Ethylene glycol bis[succinimidylsuccinate] (EGS) and m-Maleimidobenzoyl-N-hydroxysuccinimide ester (MBS) treatment were applied to plants growing in open chambers under the same media conditions as the closed chambers, but the protonemata were immobilized with 0.7% low melting point agarose. MBS and EGS were added to 30 µM and 100 µM respectively from 100× DMSO stocks. Cells were treated with the crosslinkers for 15 min before observation. For fixation and phalloidin staining, cells were processed the same way as previously reported (Vidali *et al*., 2007) but using rhodamine phalloidin (Invitrogen) instead of Alexa-488 phalloidin.

### Growth Assay

For the moss growth assay, one week old cultures of stable lines were protoplasted using established methods [11]. Plants were regenerated in top-agar (0.5% agar) in the presence of manitol for 4 days, then transferred to growth moss NH_4_ medium, PpNH_4_ (1.03 mM MgSO_4_, 1.86 mM KH_2_PO_4_, 3.3 mM Ca(NO_3_)_2_, 2.7 mM (NH_4_)_2_-tartrate, 45 µM FeSO_4_, 9.93 µM H_3_BO_3_, 220 nM CuSO_4_, 1.966 µM MnCl_2_, 231 nM CoCl_2_, 191 nM ZnSO_4_, 169 nM KI, 103 nM Na_2_MoO_4_) for 2 days and imaged. Cell walls were stained with a solution of 10 µg/ml calcofluor (fluorescent brightener 28, Sigma) in water for at least 15 min. Cellophane fragments with the cells embedded in top agar were inverted on 10 µl of staining solution, incubated for 1 min, and the cellophane was removed. An additional 10 µl of staining solution was added and mounted with a coverslip. The cells were incubated for at least 15 min and no more than 1 hr. Pictures were taken at 30× zoom with a 1× lens, as 36-bit RGB color images with a CCD camera (Leica DF300FX) on a fluorescence stereo-microscope (Leica MZ16FA). Filter combinations were for UV/DAPI setting. The blue channel of the color images, corresponding to cell wall signal was digitally separated. The resulting 12-bit image was thresholded and the total area estimated. Solidity, a morphometric parameter, was evaluated by calculating the ratio of the plant area to its convex hull area; one corresponds to a perfectly solid object and numbers smaller correspond to more branched structures. A total of 30 to 60 plants was evaluated for each replicate. All image analysis was done using macros written for ImageJ (http://rsb.info.nih.gov/ij/). Macros are available upon request. Statistical analysis was done using ANOVA and post-hoc tests in KaleidaGraph (Synergy Software).

To compare the average fluorescence per pixel to length in lily, pollen was bombarded with 3 µg of zmC13::Lifeact-mEGFP or zmC13::mEGFP. Images were collected using the 488 nm argon laser of a Nikon confocal microscope (Nikon D-Eclipse-C1) on an inverted stand (Nikon Eclipse-TE2000-S) and a 10× dry objective. Image analysis was performed using ImageJ (http://rsb.info.nih.gov/ij/). For the high resolution rate of growth analysis of lily, pollen was bombarded with 1.8 µg of zmC13::Lifeact-mEGFP then imaged 3 hours later. Pollen was imaged at 40× using the Nikon D-Eclipse-C1 microscope. Growth was tracked using MetaMorph software (Molecular Devices) and the distance from the origin was then plotted versus time. A linear regression was fit to the data and the slope represented the average velocity for the tube. To analyze the length distribution of pollen, the indicated amount of either zmC13::Lifeact-mEGFP or zmC13::mEGFP used to bombard pollen. The pollen was allowed to grow for 3 hours then fixed in 10 mM MgCl_2_, 100 mM PIPES, and 3.7% formaldehyde for 30 minutes. The pollen was then washed in LPGM, imaged and measured as described for the florescence vs. length assay. All statistical analysis was performed using Origin software (OriginLab, www.orginlab.com).

### Drug Treatments

For the analysis of Latrunculin B effects on F-actin, moss was cultured in PpNO_3_ media for 3–5 days, on top of cellophane disks. Pieces of cellophane containing protonemata were cut, flipped, and the protonemata were placed in direct contact with an agar pad containing Hoagland's medium and Latrunculin B at the indicated concentration. The cellophane was removed, 5 µl of liquid medium containing the same concentration of Latrunculin B were added, and a coverslip placed on top. The chamber was sealed with melted VALAP. Images were acquired with an interval of 10–20 min after chamber preparation. Control preparations contained DMSO at 0.2% in medium. Multiple cells and chambers were analyzed with identical results.

For the Latrunculin B sensitivity assay in moss, cells were prepared in the same way as for the growth assay (see above). Protoplasts were plated on small cellophane circles on top of agar in 96 well plates; wells were filled to the top with agar to create a flat surface to deposit the protoplasts. Cells were plated in protoplast regeneration medium in the absence of Latrunculin B for 4 days. At day 4 the cellophane discs were transferred to regular PpNH_4_ medium containing different amounts of Latrunculin B. Two days after transfer images were acquired from chlorophyll autofluorescence at a 30× zoom as 36-bit RGB color images with a CCD camera (Leica DF300FX) on a fluorescence stereo-microscope (Leica MZ16FA). Filter combinations were: excitation 480/40, dichroic 505 long pass, emission 510 long pass. The red channel of the color images, corresponding to chlorophyll fluorescence was digitally separated. The resulting 12-bit image was thresholded and the solidity estimated as mentioned above. Latrunculin treatments were performed in triplicate; a total of 7 to 38 plants was measured in each replicate. Dose response curves were fitted to the data using the sigmoidal fitting function of the program Origin (Microcal), using a logistic equation and a log10 scale for the concentration of Latrunculin B. The half maximal inhibitory concentration (IC50) was estimated from these fits. To compare the significance of the differences an ANOVA statistical test was used between the means obtained for each replicate. To calculate fractional solidity for each cell line and to plot the data, the following transformation was used: the minimum values obtained from the curve fitting were subtracted from the mean values; the resulting value was divided by the maximum value obtained by curve fitting.

For lily, bombarded pollen was grown on a slide and imaged according to our standard procedure (see above). The growth media was then replaced with fresh media plus 2 nM Latrunculin using a pipette. The procedure was performed twice to ensure that all of the media had been replaced.

## Supporting Information

Figure S1 Shows a maximal projection of the lily pollen tube shown in figure 5c. Fifteen sequential images taken in z-axis. Scale bar is 10 µm.
(0.41 MB PDF)

Movie S1 Time lapse LSCM of Lifeact-mEGFP in a moss caulonemal cell. Two frames were acquired per second. Total elapsed time was 179.5 seconds. Scale bar is 5 µm.
(3.35 MB MOV)

Movie S2 Time lapse LSCM of Nicotiana tobaccum pollen tube transformed with Lifeact-mEGFP. One frame was acquired every five seconds. Total elapsed time was 245 seconds. Scale bar is 10 µm.
(1.96 MB MOV)

Movie S3 Time lapse LSCM of Lilium formosanum pollen tube transformed with Lifeact-mEGFP. One frame was acquired every three seconds. Total elapsed time was 96 seconds. Scale bar is 10 µm.
(1.60 MB MOV)

Movie S4 Time lapse spinning disc confocal images of Lifeact-mEGFP expressing Moss caulonemal cell. One frame was acquired every two seconds. Total elapsed time was 120 seconds.
(0.55 MB MOV)

Movie S5 Time lapse LSCM DIC images of Lilium formosanum pollen tube transformed with Lifeact-mEGFP and treated with 2 nm Latrunculin B. One frame was acquired every three seconds. Total elapsed time was 246 seconds. Scale bar is 10 µm.
(15.17 MB MOV)

Movie S6 Time lapse LSCM fluorescence images of Lilium formosanum pollen tube transformed with Lifeact-mEGFP and treated with 2 nm Latrunculin B. One frame was acquired every three seconds. Total elapsed time was 246 seconds. Scale bar is 10 µm.
(15.17 MB MOV)
