# Supplementary figures and images for: Lifeact-mEGFP Reveals a Dynamic Apical F-Actin Network in Tip Growing Plant Cells
Source: PLoS One. 2009 May 29;4(5):e5744. doi: 10.1371/journal.pone.0005744 (PMC2684639; doi:10.1371/journal.pone.0005744)

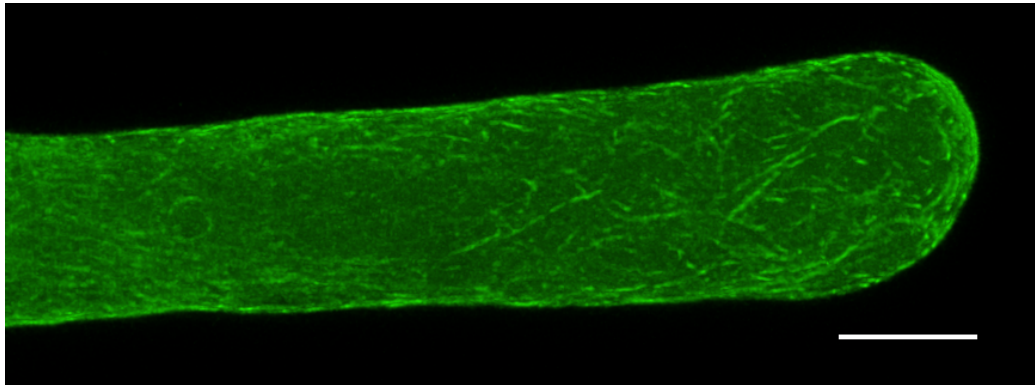

Supplemental figure 1

Supplement: Figure S1 — Shows a maximal projection of the lily pollen tube shown in figure 5c. Fifteen sequential images taken in z-axis. Scale bar is 10 µm. (0.41 MB PDF) [file pone.0005744.s001.pdf]
